# Respiratory Syncytial Virus Prophylaxis With Palivizumab Is Not Associated With Improved Lung Function in Infants of Very Low Birth Weight at Early School Age

**DOI:** 10.1016/j.chpulm.2023.100026

**Published:** 2023-11-07

**Authors:** Ingmar Fortmann, Marie-Theres Dammann, Alexander Humberg, Hannah Kraft, Alexander Herz, Kathrin Hanke, Kirstin Faust, Isabell Ricklefs, Michael Zemlin, Johannes Liese, Geraldine Engels, Christoph Härtel, Carsten Fortmann-Grote, Matthias Volkmar Kopp, Folke Brinkmann, Egbert Herting, Wolfgang Göpel, Guido Stichtenoth

**Affiliations:** a Department of Pediatrics, University of Lübeck, Lübeck, Germany; b Airway Research Center North, German Center of Lung Research (DZL), Lübeck, Germany; c Department of Pediatrics, University of Münster, Münster, Germany; d Department of General Pediatrics and Neonatology, Saarland University, Homburg, Germany; e Department of Pediatrics, University Hospital of Würzburg, Würzburg, Germany; f Department of Microbial Population Biology, Max Planck Institute for Evolutionary Biology, Plön, Germany; g Department of Paediatric Respiratory Medicine, Inselspital, University Children’s Hospital of Bern, Bern, Switzerland

**Keywords:** immunization of preterm infants, lung function, palivizumab

## Abstract

**Background:**

Prematurity and infection with respiratory syncytial virus (RSV) are major risk factors for impaired lung function beyond the neonatal period.

**Research Question:**

What are the long-term effects of palivizumab immunoprophylaxis in the first year of life on lung function and frequency of bronchitis episodes in 5- to 6-year-old preterm infants?

**Study Design and Methods:**

Preterm infants with a birth weight < 1,500 g (very low-birth weight infants [VLBWIs]) were enrolled in a German Neonatal Network cohort study between 2009 and 2016. Children were examined by a single follow-up team at 5 to 6 years of age. VLBWIs who received at least five doses of palivizumab were compared with children who never received palivizumab. Analyses were stratified by bronchopulmonary dysplasia (BPD) and gestational age. We analyzed FVC, FEV_1_, FEV_1_ to FVC ratio, and the risk of respiratory tract infections at 5 to 6 years of age via univariate analyses and linear and logistic regression models.

**Results:**

Of 1,986 VLBWIs with follow-up at 5 to 6 years of age, 951 infants (48%) received immunoprophylaxis with palivizumab. Children with BPD (n = 1,019) showed a much lower FEV_1_ than children without BPD (median FEV_1_*z* score, –1.51 vs –1.09; *P* < .001). However, FEV_1_ in children with BPD was not altered by palivizumab (median FEV_1_*z* score in 698 children with BPD who received palivizumab, –1.57 [interquartile range, –0.75 to –2.43] vs in 320 children with BPD who did not receive palivizumab, –1.37 [interquartile range, –0.69 to –2.25]; *P* = .1). As for FEV_1_, we did not find any protective effects of palivizumab for other end points or in other risk groups.

**Interpretation:**

Palivizumab immunoprophylaxis in VLBWIs is not associated with improved lung function or lower rates of respiratory tract infections in early school-age infants.

**Take-home Points**

**Study Question:** Do preterm infants benefit from seasonal immunoprophylaxis with palivizumab in terms of long-term lung function and which risk groups have the greatest benefit?

**Results:** In a cohort of 1,986 very low-birth weight infants with follow-up at 5 to 6 years of age, FEV_1_ was not altered by seasonal immunization with at least five doses of palivizumab in risk groups of preterm infants who were characterized by BPD and low gestational age.

**Interpretation:** The risk-based approach of palivizumab subscriptions according to which high-risk preterm infants are passively immunized with five injections throughout respiratory syncytial virus season is not associated with improved spirometry scores at early school age.

Very low-birth weight infants (VLBWIs) are particularly vulnerable to infectious diseases, and their vulnerability extends beyond the neonatal period into infancy, childhood, and adolescence.^1^ Premature birth, prematurity-related diseases such as bronchopulmonary dysplasia (BPD), and respiratory infections are independent risk factors for long-term respiratory impairment.^2^ Respiratory syncytial virus (RSV) is the most frequently detected pathogen of lower airway infections in infancy.^3^^,^^4^ In preterm infants, infections with RSV are associated with recurrent wheeze of early childhood,^5^ although a causal relationship remains controversial.^6^ Recurrent wheeze of early childhood has been shown to lead to long-term respiratory impairment, including reduced FEV_1_.^7^^,^^8^ Approaches that attempt to explain underlying pathomechanisms include a shift from Th2-type to Th1-type immune responses in PIs driven by early microbial exposure leading to reduced IgE sensitization and atopy risk, but simultaneously promoting sustained inflammation processes.^9^ Further hypotheses address different patterns of immunologic responses to viral infections such as eosinophilic inflammation in airway secretions, reduced interferon-γ production from mononuclear cells, and increased IL-10 secretion during convalescence, as well as disturbed airway physiologic features as a consequence of infection-related epithelial damage.^10^ However, data on long-term outcomes of early RSV infection in VLBWIs are scarce.^11^^,^^12^

Passive immunization of VLBWIs with the humanized monoclonal antibody palivizumab has been proven to reduce RSV-related hospitalization rates in PIs during the first year of life.^13^ However, the IMPACT-RSV study—a randomized, double-blinded, placebo-controlled trial—did not demonstrate effects on the total amount of days on the ICU after discharge from a primary hospital stay, duration of ventilation, or mortality,^13^ whereas long-term outcomes have not been addressed in this study. To this day, a paucity of data is available regarding long-term effects of palivizumab beyond infancy. The ongoing discussion about the cost-effectiveness of RSV prophylaxis has led international committees to limit the indication for palivizumab immunization to high-risk infants, such as preterm infants and infants with chronic lung disease or congenital heart disease. Within the group of preterm infants, the indication for or against palivizumab prophylaxis according to international guidelines, such as the committees of Austria,^14^ Sweden, Switzerland, Italy,^15^ Canada,^16^ the United States,^17^ and the United Kingdom, mainly is based on gestational age (GA), postnatal age, and the presence or absence of BPD.^3^ These recommendations predominantly rest on the increased risk of a severe course of RSV infection in infants with BPD^3^^,^^18^ and increased rehospitalization rates of extremely low-GA neonates with fewer than 28 weeks’ gestation without BPD.^16^ Data on long-term effects of palivizumab in preterm infants are limited and inconclusive, and none of the few studies that have been conducted were able—because of the low sample sizes—to stratify to risk groups targeted by immunization guidelines.^5^^,^^19^^,^^20^ Because these risk groups are characterized by a high risk of long-term impaired lung function that may be exacerbated by imbalanced immunologic responses to early infection with RSV, we hypothesized that certain GA-defined and BPD-defined VLBWI risk groups have long-term benefits resulting from seasonal RSV immunization. Accordingly, we performed an observational study in a large cohort of VLBWIs and evaluated the impact of palivizumab immunization on spirometry measurements and frequency of bronchitis episodes in risk-stratified groups of preterm infants at early school age.

## Study Design and Methods

**Figure 1:**
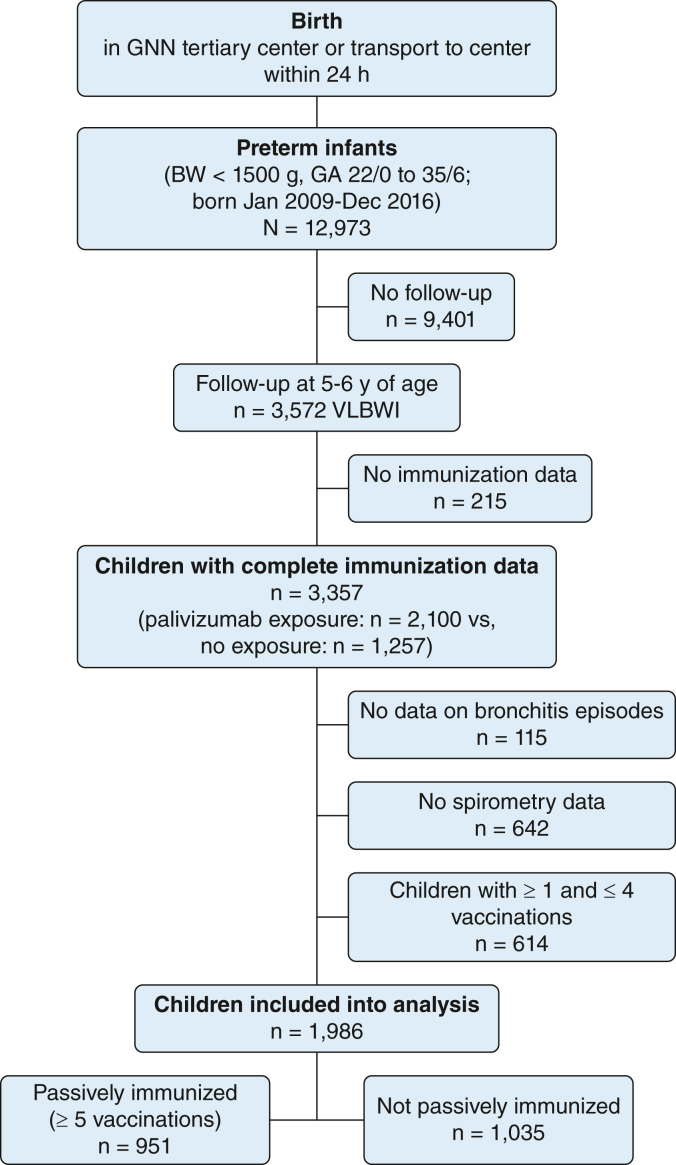
Flowchart of GNN study cohort with inclusion and exclusion criteria. Only preterm infants with a complete data set were included in the analysis of respiratory outcomes at 5 to 6 years of age. The following reasons accounted for missing spirometry data. Of the total of 642 instances of missing data, 283 spirometry examinations (44.1%) were not possible because the COVID-19 restrictions did not allow lung function tests during follow-up at that time; 94 infants (14.6%) were unable to participate because of cognitive impairment; 74 children (11.5%) faced physical limitations, an underlying disease (such as cerebral palsy [n = 33], trisomy 21 [n = 7], autism [n = 5], impairment of vision [n = 5], reduced physical resilience because of severe bronchopulmonary dysplasia [n = 5], recent exposure to a tracheal tube [n = 3], severe tracheomalacia [n = 2], muscular hypotonia [n = 2], unknown underlying disease [n = 2], recurrent laryngeal nerve paralysis [n = 1], Rubinstein-Taybi syndrome [n = 1], and recent tongue or mouth surgery [n = 1]), or both; 65 children (10.1%) did not comply with performing the test; 46 tests (7.2%) were invalid according to the judgement of the follow-up physician; 40 infants (6.2%) were not able to participate because of an upper airway infection; and in 10 cases (1.6%), technical problems occurred. In 30 instances (4.7%), the reason for not conducting spirometry was not documented. BW = birth weight; GA = gestational age; GNN = German Neonatal Network; VLBWI = very low-birth weight infant.

The German Neonatal Network (GNN; www.vlbw.de) is a population-based observational multicenter cohort study prospectively enrolling VLBWIs at 68 neonatal ICUs (NICUs) in Germany. Preterm infants with birth weight of < 1,500 g and a GA of 22 + 0 ≤ 36 + 6 weeks who were treated actively with intensive care were included. After informed written parental consent, a predefined data set on general neonatal characteristics and antenatal and postnatal treatment and outcome was recorded for each patient at the participating centers. Clinical data until discharge from the hospital were collected by each participating NICU and were monitored yearly on site by GNN staff (study nurse or pediatrician trained in neonatology). After monitoring, the patient records were sent to the study center (Lübeck), where data were coded and evaluated. For this study, data were collected from infants born between January 1, 2009, and December 31, 2016. Details on patient selection are given in Figure 1.

At 5 to 6 years of age, GNN-participating children were invited to a follow-up assessment by the study office. Within the invitation procedure, the study team contacted the clinic of birth for possible onsite follow-up examination dates. The contact database was searched randomly for possible candidates with focus set on VLBWIs born at fewer than 28 weeks of gestation, but children born at more than 28 weeks ofgestation were not necessarily excluded. Preterm infants who were born in the clinic of interest whose parents could be reached by telephone or post and were able to attend the follow-up appointment received an invitation letter. The procedure for invitation was identical for all participating centers. Children were assessed on site by a single GNN follow-up team blinded to any interventions or complications during primary NICU stay. Reproducibility of the results was ensured further by using identical instruments or equipment for all children, which were brought to the study sites by the GNN team. The follow-up included the performance of a hearing test, a visual screening, and spirometry. Parents were asked to answer questions concerning medical history and current medical needs, and detailed information on the children’s social background, illnesses, and general development and behavior. Data on the children’s vaccination history were generated by copying the children’s vaccination certificates in which all vaccinations that the child has received are documented (inpatient and outpatient vaccinations). Type of vaccination, date of vaccination, and number of immunizations were entered into the database. Apart from onsite follow-up, a questionnaire regarding the children’s health status was sent to the families on a yearly basis after discharge from the primary NICU stay.

Spirometry was performed with an EasyOne spirometer (NDD Medizintechnik AG) by the study team’s physician at all sites. Although the maximum number of attempts was 10, the physician who supervised the performance of the spirometry assessed the quality of the spirometry evaluating the volume-time curve and flow-volume curve of each test. In addition, the program includes an integrated technical validation of the volume loops. This way, only results of sufficient quality were used, whereas the test with the highest quality was selected for analysis. Interactive computer graphics were used to motivate the children for spirometry (ie, blowing up a balloon, blowing leaves from a tree). FEV_1_ and FVC were documented in liters and were analyzed as % predicted values based on a European reference population.^21^ In addition, the FEV_1_ and FVC *z* scores were calculated according to the Global Lung Function Initiative.^22^ An FEV_1_ of < 80% was defined as an additional outcome parameter of our study because it was used frequently in observational reports.^23^ The Tiffeneau index, defined as the ratio of FEV_1_ to FVC, was calculated because sometimes disynaptic growth and mild lung obstruction are seen in infants with BPD and—if very mild—may not be diagnosed by a reduction in FEV_1_. The parental questionnaires, as part of the GNN follow-up, included information on the frequency of bronchitis within the last year, which served as secondary respiratory outcome of this study.

The German guidelines on RSV prophylaxis (valid until August 2023 and currently being updated) recommend the application of five intramuscular palivizumab doses during the RSV season at intervals of 4 weeks.^3^ The 4-week intervals should be observed strictly, especially at the beginning of the immunoprophylaxis. Preterm infants younger than 24 months who were treated for severe BPD with supplemental oxygen, with a ventilator, or both within the 3 months before the start of the RSV season (November) are recommended to receive immunoprophylaxis with palivizumab.^3^ Infants younger than 6 months and of fewer than 28 + 6 weeks of gestation optionally can be immunized. Preterm infants between 29 + 0 and 34 + 6 weeks’ gestation can be indicated (no general recommendation) for immunoprophylaxis with palivizumab under strict consideration of additional risk factors (discharge into RSV season, severe underlying neurologic disease, nursery attendance, or siblings in external childcare). Palivizumab is not indicated to prevent nosocomial RSV infection. International guidelines base their recommendations on palivizumab in preterm infants mainly on the presence or absence of BPD, GA, and postnatal age.^14^, ^15^, ^16^

### Definitions

GA was calculated from the best obstetric estimate based on early prenatal ultrasound and obstetric examination. Small for GA was defined as a birth weight less than the 10th percentile for GA according to sex-specific standards for birth weight by GA in Germany.^24^ According to the definition used in the German guideline on RSV prophylaxis,^3^ BPD was divided into two grades: moderate or severe BPD was defined as supplemental oxygen or ventilatory support at 36 weeks’ postmenstrual age and mild BPD was defined as supplemental oxygen or ventilatory support at 28 days of age but not at 36 weeks’ postmenstrual age.^18^^,^^25^ The bronchitis risk at 5 to 6 years of age was defined by the number of bronchitis episodes within the last year, measured by a parental questionnaire that was filled out at the follow-up visit. RSV season was defined as between November 1 and March 31, based on surveillance data of the Centers for Disease Control and Prevention.^3^

### Ethics

All study parts were approved by the University of Lübeck Ethical Committee and the committees of the participating centers (Identifier: 08-022; date of approval: June 27, 2008). Informed consent was obtained from all parents.

### Statistical Analysis

Data were analyzed with SPSS Statistics version 28.0 software (IBM). The global type I error level was set to 0.05. All *P* values given are two-sided. Incomplete data sets were not included (Fig 1). To address the effects of RSV prophylaxis, we compared infants with seasonal RSV prophylaxis (at least five doses of palivizumab) with unimmunized infants. Subsequent analyses were performed by stratifying to the main criteria considered for indication of palivizumab, which are BPD and GA.

We used multivariate logistic and linear regression models and known outcome confounders as independent variables: GA per week, multiple birth, blood culture-positive sepsis,^1^ BPD,^26^ nicotine exposure, elder siblings, discharge into RSV season, immunization against influenza, and feeding with human milk at NICU discharge.^27^ Notably, FEV_1_ *z* scores already are adjusted for important confounders such as sex and postnatal age.^21^^,^^22^ ORs and 95% CIs were calculated to identify the effect of RSV prophylaxis on outcomes. To address the problem of multiple comparisons, we performed Bonferroni corrections for multivariate analyses to protect from statistical type I errors. Additional information derived from Bonferroni correction is indicated in the tables respectively. Table 2

**Table 1:** Clinical Characteristics of Study Cohort Stratified by Passive RSV Immunization Status

Characteristic	Not Passively Immunized (n = 1,035; 52.1%)	Passively Immunized (n = 951; 47.9%)	*P* Value^a^	Total (N = 1,986)
Gestational age, wk, median (IQR)	29.6 (28.3-30.9)	27.1 (25.6-28.6)	< .001^b^	28.4 (26.7-30.0)
Birth weight, g, median (IQR)	1,200 (980-1,370)	900 (706-1,120)	< .001^b^	1,050 (820-1,290)
Sex, male, % (95% CI)	49.1 (46.0-52.1)	51.3 (48.1-54.5)	.32	50.1 (48.0-52.3)
Multiple births, % (95% CI)	39.3 (36.4-42.3)	37.2 (34.2-40.3)	.33	38.3 (36.2-40.5)
Vaginal birth, % (95% CI)	8.2 (6.7-10.0)	10.2 (8.4-12.3)	.03	9.2 (8.0-10.5)
Discharge into (pre-)RSV season (September-March), % (95% CI)	49.8 (46.7-52.8)	52.2 (50.0-56.4)	.1	51.4 (49.2-53.6)
Immunization against influenza (any exposure), % (95% CI)	14.0 (12.8-17.2)	30.6 (28.0-33.2)	< .001	23.3 (21.6-25.1)
Hexavalent and pneumococcal immunizations	99.3 (98.7-99.7)	99.8 (99.3-100)	.13	99.5 (99.2-99.8)
Outcomes, % (95% CI)				
Mild BPD	25.1 (22.6-27.9)	48.7 (45.5-51.9)	< .001	36.4 (34.2-28.6)
Moderate to severe BPD	5.8 (4.5-7.4)	24.7 (22.0-27.5)	< .001	14.9 (13.3-16.5)

BPD = bronchopulmonary dysplasia; IQR = interquartile range; RSV = respiratory syncytial virus.

a *P* values for univariate analyses were derived from the χ^2^ test, if not otherwise indicated.

b Mann-Whitney *U* test.

**Table 2:** Clinical Characteristics of Subcohort of Infants of Fewer Than 29 Weeks’ Gestation Stratified by Passive RSV Immunization Status

Characteristic	Not Passively Immunized (n = 360; 32.1%)	Passively Immunized (n = 761; 67.9%)	*P* Value^a^	Total (N = 1,121)
Gestational age, wk, median (IQR)	27.6 (26.3-28.9)	26.6 (25.1-27.9)	< .001^b^	27.0 (25.4-28.0)
Birth weight, g, median (IQR)	980 (745-1,130)	840 (666-990)	< .001^b^	870 (690-1,050)
Sex, male, % (95% CI)	50.6 (45.4-55.7)	50.6 (47.0-54.1)	.99	50.6 (47.7-53.3)
Multiple births, % (95% CI)	35.0 (30.2-40.0)	35.7 (32.4-39.2)	.81	35.5 (32.7-38.3)
Vaginal birth, % (95% CI)	12.8 (9.6-16.5)	11.1 (9.0-13.5)	.57	11.6 (9.9-13.6)
Discharge into (pre-)RSV season (September-March), % (95% CI)	43.9 (38.8-49.0)	53.7 (50.2-57.3)	.002	50.6 (47.7-53.5)
Outcomes, % (95% CI)				
Mild BPD	53.6 (48.4-58.7)	57.3 (53.8-60.8)	< .001	56.1 (53.2-59.0)
Moderate to severe BPD	11.9 (8.9-15.6)	28.1 (25.0-31.4)	< .001	22.9 (20.5-25.5)

BPD = bronchopulmonary dysplasia; IQR = interquartile range; RSV = respiratory syncytial virus.

a *P* values for univariate analyses were derived from the χ^2^ test, if not otherwise indicated.

b Mann-Whitney *U* test.

## Results

Within the study period from January 1, 2009, through December 31, 2016, 12,973 VLBWIs were discharged in 63 participating GNN sites. Detailed information on included and excluded VLBWIs are given in the study flow chart (Fig 1). For 3,572 VLBWIs, follow-up investigations were performed. When compared with VLBWIs who were not followed up, infants with follow-up were characterized by a lower median GA (28.2 weeks [interquartile range (IQR), 26.3-29.7 weeks] vs 29.1 weeks [IQR, 26.9-31.0 weeks]; *P* < .001) and lower birth weight (990 g [IQR, 775-1,240 g] vs 1,130 g [IQR, 850-1,360 g]; *P* < .001), and multiple birth was overrepresented in the follow-up cohort. Two hundred fifteen children at follow-up did not provide vaccination cards or data on RSV immunization was incomplete. Of 3,357 VLBWIs with detailed information on the history of immunizations, 2,100 children had received at least one dose of palivizumab, whereas 1,257 children never received immunoprophylaxis against RSV. For the analysis of respiratory outcomes at early school age, we included only children with a complete data set, so that 115 children with missing information on the number of bronchitis episodes and 642 children without spirometry data were excluded. Detailed information on children with missing spirometry data can be found in the flowchart caption of Figure 1. When compared with the group of children with spirometry data, those without such data were characterized by a lower median GA (27.4 weeks [IQR, 25.6-29.6 weeks] vs 28.3 weeks [IQR, 26.6-29.7 weeks]; *P* < .001) and lower birth weight (920 g [IQR, 680-1,190 g] vs 993 g [IQR, 880-1,245 g]; *P* < .001), whereas they were not significantly different in other baseline characteristics such as delivery mode, sex, and multiple births. For the analysis of infants receiving seasonal immunoprophylaxis (at least five doses) compared with infants not treated with palivizumab, we excluded 614 VLBWIs who received fewer than five but not zero doses of palivizumab. Finally, 1,986 VLBWIs met the inclusion criteria for respiratory end points and were stratified in two groups: 951 VLBWI were immunized passively with at least five doses of palivizumab, whereas 1,035 VLBWIs did not receive any dose of palivizumab.

**Figure 2:**
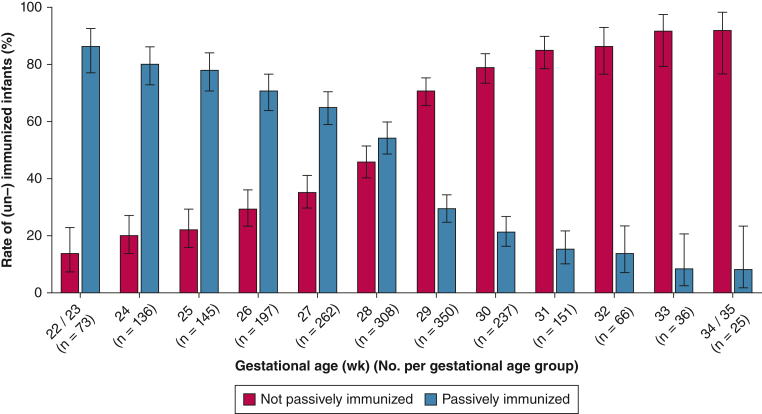
Graph showing the frequency of passive respiratory syncytial virus (RSV) immunization (at least five doses of palivizumab, indicated in blue) vs no immunization (zero doses of palivizumab, indicated in red) in Germany stratified by gestational age groups. Error bars show the 95% CIs. Infants younger than 26 weeks of gestation are exposed to a complete course of RSV prophylaxis in more than 80% of cases; infants of more than 28 weeks’ gestation largely are not immunized (69.6%).

**e-Fig 1:**
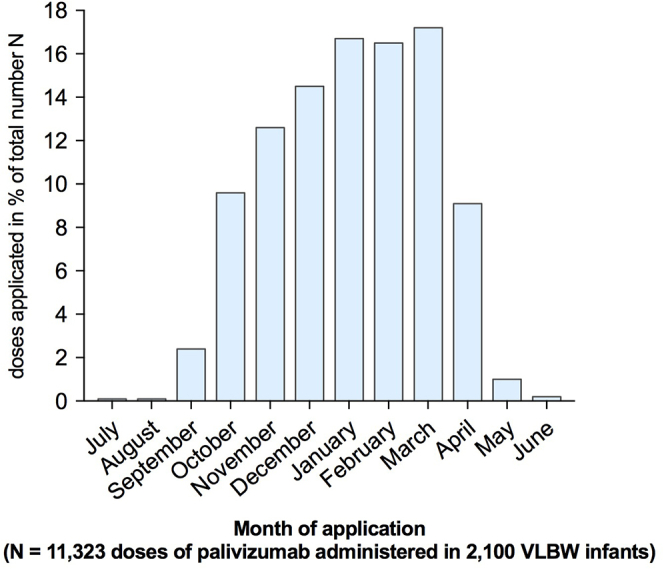


**e-Fig 2:**
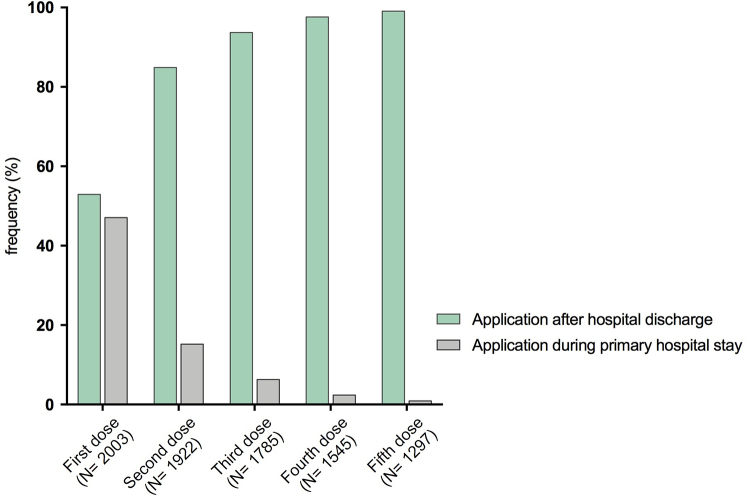


Infants with exposure to palivizumab were immunized with a median of 5.0 doses (IQR, 3-7 doses; 5th-95th range, 1-11 doses). Most palivizumab doses (77.5%) were administered during RSV season (e-Fig 1). Almost one-half of the infants’ first passive immunizations (47.1%) and 15.2% of secondary applications were performed during primary NICU stay (e-Fig 2). For the intervals between the first, second, and third application of palivizumab, which are recommended to be observed strictly, we measured a median of 29 days (IQR, 28.0-33.0 days) for the first and second interval. Notably, 25% of the second and third immunizations were administered on day 33 or later after the previous dose. As shown in Figure 2, we noted a strong negative correlation between GA and the palivizumab immunization rate in Germany. Infants of fewer than 26 weeks’ gestation are exposed to a complete course of RSV prophylaxis in > 80% of cases, whereas infants of more than 28 weeks of gestation are largely (69.6%) unimmunized.

### Vulnerability of Palivizumab-Exposed Infants

Baseline clinical characteristics of the study cohort are shown in Table 1 and include a median gestational age of 28.4 weeks and a median birth weight of 1,050 g. Passively immunized infants had a lower gestational age (median, 27.1 weeks vs 29.6 weeks) and birth weight (median, 900 g vs 1,200 g) compared with nonimmunized VLBWIs and, as a consequence, showed a higher rate of adverse short-term outcomes such as BPD. No remarkable differences were identified between the subgroups in terms of further baseline clinical characteristics (Table 1). Infants at the follow-up visit were a median of 5.4 years of age (IQR, 5.25-5.83 years), whereas age at follow-up was distributed equally across the subgroups of immunized and unimmunized children (5.54 years [IQR, 5.29-5.87 years] vs 5.54 years [IQR, 5.23-5.82 years]; *P* = .67).

### Palivizumab and Long-term Respiratory Function

**Figure 3:**
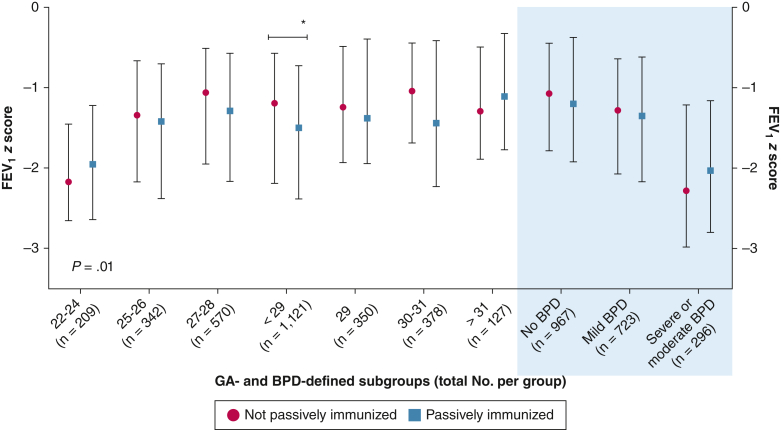
Graph showing FEV_1_*z* scores stratified to exposure to respiratory syncytial virus (RSV) prophylaxis vs no RSV immunization in GA-defined and BPD-defined subgroups. Point plots show median and error bars show interquartile ranges. P values were derived from the Mann-Whitney U test. ∗Significance level, *P* < .05. Group sizes of GA groups : n = 209 children between 22 and 24 weeks’ gestation (37 nonimmunized vs 172 immunized); n = 342 children between 25 and 26 weeks’ gestation (90 nonimmunized vs 252 immunized); n = 570 children between 27 and 28 weeks’ gestation (233 nonimmunized vs 337 immunized); n = 1,121 children of < 29 weeks’ gestation (360 nonimmunized vs 761 immunized); n = 378 children of 29 weeks’ gestation (247 nonimmunized vs 103 immunized); n = 378 children between 30 and 31 weeks’ gestation (315 nonimmunized vs 73 immunized); and n = 127 children of > 31 weeks’ gestation (113 nonimmunized vs 14 immunized). Group sizes of BPD groups: n = 967 children without BPD (714 nonimmunized vs 253 immunized); n = 723 children with mild BPD (260 nonimmunized vs 463 immunized); and n = 296 children with moderate or severe BPD (61 nonimmunized vs 235 immunized). BPD = bronchopulmonary dysplasia; GA = gestational age.

**e-Fig 3:**
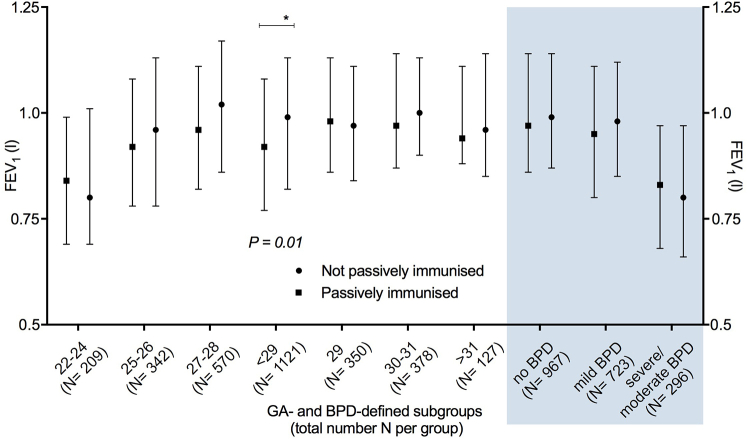


Univariate analyses of spirometry measures at 5 to 6 years of age in narrowly defined GA groups did not show significant differences in FEV_1_ *z* scores between children who received seasonal immunoprophylaxis and those in the nonimmunized group (Fig 3) (raw data of FEV_1_ in liters shown in e-Fig 3). Likewise, no group differences were found in other spirometry measures such as FVC and FEV_1_ to FVC ratio (*z* scores and raw data; data not shown). Notably, nonimmunized infants in the subgroup of those with fewer than 29 weeks’ gestation (n = 1,121) displayed an improved median FEV_1_ and FVC. However, when adjusted for confounders of lung function impairment, we did not observe FEV_1_-improving signals of palivizumab administration in any gestational subgroup (Table 2).

**e-Fig 4:**
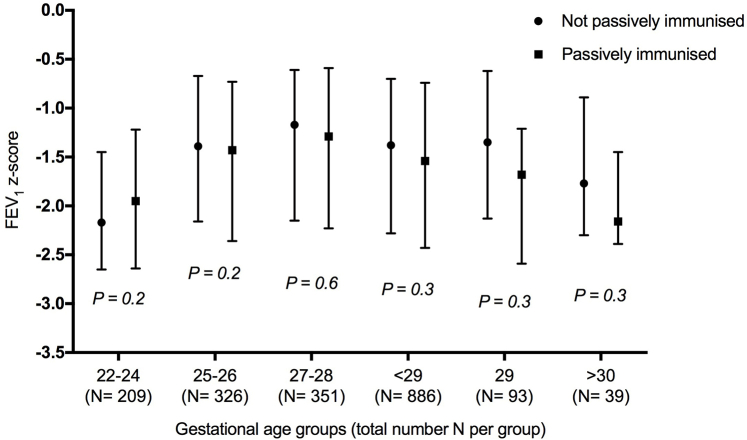


Further, we observed no lung function differences for infants with different BPD severity stages stratified to seasonal palivizumab administration (Fig 3) and no immunization-related benefit in the most susceptible infants characterized by both BPD and extreme prematurity (e-Fig 4). In the subgroup of infants with BPD (n = 1,018 VLBWIs), immunized children were characterized by lower GA (26.4 weeks [IQR, 25.0-27.7 weeks] vs 27.6 weeks [IQR, 26.2-29.0 weeks]; *P* < .001) and lower birth weight (810 g [IQR, 650-980 g] vs 942 g [IQR, 740-1,103 g]; *P* < .001), whereas they did not differ in further baseline clinical characteristics. However, when adjusted to these confounders, our linear and logistic regression models did not demonstrate any immunization-related associations with FEV_1_ *z* scores in infants with BPD (Table 2).

Regarding the secondary outcome, we observed no significant differences in the rate of bronchitis episodes during the last year before follow-up stratified to palivizumab administration and regardless of stratification to GA- or BPD-related subgroups (e-Table 1). Within the GA- and BPD-defined risk subgroups, our logistic regression model did not show any independent effects of palivizumab immunization on the bronchitis risk when adjusted for known confounders (Table 2).

### VLBWIs of Fewer Than 29 Weeks’ Gestation

In the subgroup analysis of infants of fewer than 29 weeks’ gestation, we aimed at reducing differences in clinical baseline characteristics such as GA and birth weight (Table 3). Seven hundred sixty-one extremely low-GA neonates (67.9%) received seasonal RSV immunization, whereas 360 extremely low-GA neonates (32.1%) were not immunized. As for FEV_1_, we did not find any protective effects of palivizumab for our end points in any risk group defined by BPD, low GA, or both; that is, we noted no independent association between seasonal palivizumab administration and FEV_1_ *z* score (effect size β, –0.1; 95% CI, –0.3 to 0.2; *P* = .4) or the bronchitis risk at early school age (OR, 0.9; 95% CI, –0.6 to 1.4; *P* = .6).

**Table 3:** Effects of Passive Immunization With Palivizumab (Five or More Doses) on Bronchitis Risk at 5 to 6 Years of Age, FEV_1_ < 80%, and *z* Scores of FEV_1_ in GA-Defined and BPD-Defined Risk Groups

Subgroup	Adjusted OR, FEV_1_ < 80%^a^	Adjusted Effect Size β, FEV_1_*z* Score^b^	Adjusted OR, Bronchitis Risk^a^
GA, wk			
22-24	0.4 (0.1-1.7)	–0.1 (–2.2 to 0.7)	0.1 (0.02-0.5)
P value	.2	.3	.01^c^
No. of patients^d^	113	105	112
25-26	0.6 (0.2-1.5)187	0.01 (–0.7 to 0.7)	0.6 (0.3-1.5)
P value	.3	.9	.3
No. of patients	187	169	185
27-28	1.0 (0.5-1.9)	–0.1 (–0.6 to 0.2)	1.1 (0.6-2.0)
P value	.9	.4	.7
No. of patients	332	299	330
29^d^	1.4 (0.6-3.2)	–0.1 (–0.8 to 0.3)	0.9 (0.4-1.9)
P value	.5	.3	.8
No. of patients	204	200	201
< 29^e^	0.8 (0.5-1.3)	–0.1 (–0.5 to 0.2)	0.9 (0.6-1.4)
P value	.4	.4	.6
No. of patients	632	620	627
30-31	1.9 (0.6-5.4)	–0.3 (–2.1 to 0.2)	1.6 (0.7-4.0)
P value	.3	.1	.3
No. of patients	175	180	173
> 31	0.9 (0.1-17.1)	0.1 (–2.2 to 5.6)	1.0 (0.8-1.2)
P value	.9	.9	.9
No. of patients	53	41	53
BPD			
Mild	1.1 (0.6-2.0)	–0.1 (–0.6 to 0.7)	0.9 (0.5-1.6)
P value	.7	.3	.8
No. of patients	417	366	412
Moderate to severe	0.6 (0.3-1.5)	–0.1 (–1.3 to 0.7)	1.4 (0.6-3.7)
P value	.3	.5	.4
No. of patients	193	175	181

Data are presented as OR (95% CI) or β (95% CI), unless otherwise indicated. Logistic and linear regression models were carried out using GA, sex, multiple births, blood culture-positive sepsis, discharge into respiratory syncytial virus season, immunization against influenza, nicotine exposure, older siblings in household, feeding human milk at hospital discharge, and passive immunization with palivizumab as independent variables. BPD = bronchopulmonary dysplasia; GA = gestational age.

a BPD was included additionally in the models for GA subgroups.

b The model for FEV_1_*z* scores were not adjusted for sex because *z* scores already are adjusted.

c Does not reach significant level after Bonferroni correction (multiple testing).

d No. included in the model.

e The model for 29 wk was not corrected for GA per wk.

### Sensitivity Analysis

In our analysis, we compared immunized children (at least five doses) with VLBWIs who had not received any dose of palivizumab. For that reason, 614 infants who received between one and four doses of palivizumab were not included in the analysis (Fig 1). This approach was chosen to distinguish clearly between a group with a relevant exposure to palivizumab and another group with no exposure at all. To evaluate the bias that may have been introduced by our definition of the variable *passively immunized*, we performed a sensitivity analysis. We reperformed our calculations by defining the cohort of passively immunized infants as children who had received at least two doses of palivizumab (n = 1,452). Consistent with our results in Figure 3 and Table 2, univariate analyses and regression models did not reveal any associations between palivizumab and our end points, that is, for FEV_1_ *z* scores in the group of passively immunized children of fewer than 29 weeks’ gestation: 360 nonimmunized children vs 1,127 passively immunized preterm children: –1.19 (IQR, –2.19 to –0.57) vs –1.48 (IQR, –2.38 to –0.71; *P* = .06). The same results were reproduced for children at risk of long-term respiratory impairment because of moderate or severe BPD (ie, 61 nonimmunized children vs 342 passively immunized children, –2.28 [IQR, –2.98 to –1.21] vs –2.01 [IQR, –2.79 to –1.13]; *P* = .07).

In line with our results in Table 2 we did not observe an independent effect of exposure to at least two doses of palivizumab on long-term respiratory outcomes when adjusting the model for the previously mentioned confounders (ie, linear regression model for FEV_1_ *z* scores within the subcohort of preterm children of fewer than 29 weeks’ gestation: effect size β, 0.03 [95% CI, –0.12 to 0.25]; *P* = .5) or logistic regression for FEV < 80% (< 29 weeks’ gestation): OR, 0.9 [95% CI, 0.4-1.9]; *P* = .8). In the BPD-defined risk groups, we likewise observed no independent effect. The linear regression model for FEV_1_ *z* scores within the subcohort of preterm children with severe BPD showed an effect size β of 0.03 (95% CI, –0.3 to 0.5; *P* = .7) and the logistic regression for FEV of < 80% was as follows: OR, 1.4 (95% CI, 0.3-6.7; *P* = .7).

### Outcomes at 12 to 24 Months of Age

Questionnaires regarding the infants’ health state that were mailed to the families at 12 to 24 months of age showed a relatively low return rate. Infants in the group of children who were not passively immunized (n = 1,035) were in the hospital for a mean ± SD of 6.21 ± 21.9 nights between 12 and 24 months of age as compared with infants with at least five doses of palivizumab, who spent a mean ± SD of 11.3 ± 34.6 nights in hospital during the same age (*P* = .001). In a linear regression model adjusted for GA, sex, multiple birth, and BPD, the number of hospital nights per year (age 12-24 months) was not associated with exposure to any dose of palivizumab (*P* = .8), but mainly with GA (*P* < .001). However, these questionnaire-based data were available only from 764 infants (369 unimmunized infants vs 395 immunized infants), which reflects fewer than one-half of the infants included in our analysis. The same applies for the number of bronchitis episodes within the 12- to 24-months age range (mean ± SD, 0.8 ± 1.44 [n = 505 unimmunized children] vs 1.0 ± 1.86 [n = 521 immunized children]; *P* = .6; total n = 1,026 children).

## Discussion

To our knowledge, we present the largest population-based cohort study on long-term respiratory outcome effects after palivizumab immunoprophylaxis in high-risk preterm children.^5^^,^^19^^,^^20^ We hypothesized that infants with low GA, BPD, or both would benefit from complete seasonal RSV immunization in terms of long-term respiratory function by being protected from severe early RSV infection. The research question is of high relevance because low GA and BPD are among the main risk factors considered when indicating palivizumab in VLBWIs, although a paucity of data is available on long-term outcomes to justify this strategy.^3^ Our study demonstrates a high BPD rate of almost 25% in infants who received palivizumab. Children with BPD showed a much lower FEV_1_ than children without BPD; however, FEV_1_ in children with BPD was not altered by palivizumab. The recommended risk-based approach for passive immunizations has been predicated on improved short-term outcomes such as reduced severity of disease and decreased hospitalization rates in the first year of life.^13^ However, the strategy remains controversial because of high costs, lacking effects on mortality or ventilation rates, and because of limited data on long-term benefits. Our observation is in line with previously published data by Amitai et al,^19^ who found no improvement of FEV_1_ in 17 infants born after the implementation of palivizumab immunization when compared with a control group born before palivizumab vaccinations were performed. Similarly, Prais et al^5^ presented a cohort of 30 immunized preterm infants at a mean age of 8.9 years who did not significantly differ in terms of lung function measures when compared with age-matched control infants. The only study suggesting long-term beneficial effects of palivizumab on lung function at 7 to 10 years of age was limited by a very small sample size of only 13 preterm infants.^20^ Within all studies addressing long-term outcomes of preterm infants—including ours—it must be acknowledged that a variety of residual confounding factors potentially affect outcome data that are difficult to adjust for in the complex context of child development after preterm birth. Our data confirm that preterm infants for whom immunization with palivizumab is indicated represent a particularly vulnerable group of infants characterized by extreme prematurity and high rates of severe complications such as BPD. For this reason, nonimmunized PIs may have even better spirometry scores (subgroup of those of fewer than 29 weeks’ GA), reflecting lower overall susceptibility in infants without indication for immunoprophylaxis. However, when compared with previous studies, our analysis adds stratification and adjustment to the main risk factors of preterm infants for long-term respiratory impairment (GA and BPD), which often are considered in the clinical decision of whether to administer palivizumab. Finally, seasonal immunization with palivizumab was not associated with altered lung function measurements at early school age after adjustment for relevant confounders, whereas a causal relationship cannot be drawn because of the observational design of the study.

Acute bronchiolitis and infection with RSV in early life have been suggested to be associated with an increased risk of asthma development^28^, ^29^, ^30^ up to 11 years of age.^30^ However, a causal relationship of these associations remains unclear. That the prevention of RSV infection through palivizumab reduces the incidence of recurrent wheeze of early childhood also has been suggested for preterm infants of different age groups and with different predispositions, such as family history of asthma or atopy.^31^, ^32^, ^33^ However, the most vulnerable infants of fewer than 29 weeks’ gestation largely have been neglected and most trials focus on the first 2 years of life. A recent World Health Organization report from 2020 concluded that the evidence for establishing a causal association between RSV lower respiratory tract infection and recurrent wheeze of early childhood or asthma is inconclusive, and that RSV monoclonal antibodies do not have a substantial effect on theses outcomes.^6^ However, our hypothesis of a protective effect on lung function of passive immunization with palivizumab is based on multiple association studies and on the widely held assumption that this association reflects a causal relationship, which may influence the clinician’s decision to subscribe palivizumab. In line with the World Health Organization report’s conclusions, our observational data did not find a difference between passively immunized and nonimmunized infants regarding the occurrence of any bronchitis at early school age, even if adjusted for known confounders. Notably, our study group previously reported a 10% incidence reduction of bronchitis episodes in VLBWIs at 1 years and 5 to 6 years of age after early immunization with palivizumab and routine vaccinations.^34^^,^^35^ However, timely administered routine immunizations can enhance trained immunity, which may result in decreased overall susceptibility to infections during infancy and childhood.^35^ Nevertheless, a paucity of data is available on immunization rates of preterm infants, not only in Germany. We illustrate the corresponding data from the GNN, which demonstrate that significant rates of immunized and unimmunized infants exist across all GA groups. Herewith, our data reflect the international controversy that exists regarding immunoprophylaxis with palivizumab in preterm infants. For that reason and given the very high costs of palivizumab, it is important to integrate the results of long-term studies into the elaboration of new guidelines or when considering the need for new vaccines.

### Strengths and Limitations

The main strengths of this study are the large sample size and the standardized onsite follow-up examinations that were performed by a single study team masked to the children’s group assignment. Among the limitations is the lack of information on the occurrence of RSV disease within the study cohort because RSV exposure might not have been equal across the subgroups. Further, the risk of bronchitis is based on parental questionnaires, limited to the fifth to sixth year of life, and not on physical examination findings at the time of infection. Because of the observational design of the GNN, we cannot present data on bronchodilator reversibility or skin test data, and further confounders may exist such as antibiotic exposure, gut dysbiosis, nutrition and growth, use of various drugs, severe infection, or unrecognized systemic inflammatory reactions that might have an impact on developmental and pulmonary outcomes for which we cannot adjust.^1^ As in previous trials, our study likewise describes associations and cannot conclude causal relationships because it is a post hoc analysis of an observational population-based design. The associated problem of multivariate testing was addressed by using Bonferroni correction, which reduces the probability of committing a type I error, while the likelihood of committing a type II error is heightened at the same time. Studies on pulmonary function in premature children are always limited by survival bias, because only children with normal enough development to undergo pulmonary function tests are included. Further, our follow-up strategy does not rule out a risk of selection bias, so that we are not able to reduce differences in baseline clinical characteristics between infants with and without follow-up. Some factors that are associated with improved long-term lung function were more common in children who were followed up, including higher GA and birth weight. Infants with missing spirometry data were more immature and smaller than infants with lung function tests. These differences were accounted for by stratifying into narrowly defined subgroups of GA in univariate statistics and adjusting for confounding factors in multivariate logistic regression models. Finally, forced midexpiratory flow is a most sensitive measure of airflow in peripheral airways where primary airflow obstruction originates, and it is reduced in early bronchial impairment.^36^ Future studies should include forced midexpiratory flow because it may be an early predictor of COPD.^37^^,^^38^

## Interpretation

The risk-based approach of palivizumab prescriptions in preterm infants, according to which subgroups at high risk of respiratory impairment are passively immunized with five injections given monthly throughout RSV season, is not associated with improved spirometry scores at early school age. New antibodies like nirsevimab and active immunization with RSV prefusion F protein vaccine have been attracting attention recently and are likely to substitute palivizumab immunoprophylaxis soon. Our findings emphasize that recommendations for these new drugs should be based on outcomes used as end points in controlled trials, rather than presumed long-term benefits that have never been validated.

## Funding/Support

The German Neonatal Network is funded by the German Ministry for Education and Research (GNN grant No. 01ER0805 and 01ER1501). No payment, honorarium, grant or other form of payment has been given to the authors.

## Financial/Nonfinancial Disclosures

The authors have reported to *CHEST Pulmonary* the following: E. H. and J. L. are participants in a European study on the burden of RSV (the Burden of RSV Infection in Young Children in European Countries (BRICE) study). E. H. served as an advisor for the German and the European parent association concerning information material about RSV for families. These organizations received support from Sanofi-Aventis Deutschland GmbH, AbbVie, Inc., and AstraZeneca GmbH. E. H. and W. G. are participants in an international study on the use of novel RSV antibodies. This study and the BRICE study are supported by Merck Sharp & Dohme. E. H., W. G., and J. L. received only institutional support, and no personal financial support. E. H. and J. L. are members of the guideline committee on “Prevention of Severe RSV Infection” (AWMF, Arbeitsgemeinschaft der Wissenschaftlichen medizinischen Fachgesellschaften, 048-012). M. V. K. has received research grants from the Bundesministerium für Bildung & Forschung grant for the German Center of Lung Research (Deutsches Zentrum für Lungenfroschung) and consulting and speakers fees from Allergopharma GmbH, Sanofi-Aventis GmbH, Chiesi GmbH, and Infectopharm GmbH. None declared (I. F., M.-T. D., A. Humberg, H. K., A. Herz, K. H., K. F., I. R., M. Z., G. E., C. H., C. F.-G., F. B., G. S.).

## Supplementary Data

e-Online Data
