# Supplementary material for: Respiratory Syncytial Virus Prophylaxis With Palivizumab Is Not Associated With Improved Lung Function in Infants of Very Low Birth Weight at Early School Age
Source: CHEST Pulm. 2023 Nov 7;2(1):100026. doi: 10.1016/j.chpulm.2023.100026 (PMC13418319; doi:10.1016/j.chpulm.2023.100026)
Supplement: e-Online Data [file mmc1.docx]

**e-Table 1**. Bronchitis episodes within last year before follow-up stratified to passive RSV immunisation and risk groups defined by gestational age and BPD

|  | **Not passively**  **immunised**  (*N* = 1035; 52.1%) | **Passively immunised**  (*N* = 951; 47.9%) |  | **total**  (*N =* 1986) |
| --- | --- | --- | --- | --- |
| **Subgroups (GA)** | **Bronchitis episodes (%)**  **number *N***  **%** | | **p** | **%**  **Number *N*** |
| **GA 22-24 weeks** | 27.0  *N* = 10/37 | 23.8  *N* = 41/172 | .6 | 24.4  *N* = 51/209 |
| **GA 25-26 weeks** | 23.9  *N* = 21/88 | 26.8  *N* =67/250 | .2 | 26.0  *N* = 88/338 |
| **GA 27-28 weeks** | 19.7  *N* = 46/233 | 24.8  *N* = 83/335 | .7 | 22.7  *N* = 129/568 |
| **GA 29 weeks** | 21.1  *N* = 52/246 | 28.7  *N* = 29/101 | .1 | 23.3  *N* = 81/347 |
| **GA <29 weeks** | 21.5  *N* = 77/358 | 25.2  *N* = 191/757 | .1 | 22.7  *N* = 268/1115 |
| **GA 30-31 weeks** | 20.8  *N* = 65/313 | 24.7  *N* = 18/73 | .4 | 21.5  *N* = 83/386 |
| **GA >31 weeks** | 15.0  *N* = 17/113 | 7.1  *N* = 1/14 | .4 | 14.2  *N* = 18/127 |
| **Subgroups (BPD)** | **Bronchitis episodes (%)**  **number *N*** | | **p** | **%**  **Number** ***N*** |
| **mild BPD** | 18.1  *N* = 47/260 | 23.3  *N* = 107/459 | .1 | 21.4  *N* = 154/719 |
| **Moderate / severe BPD** | 34.5  *N* = 20/58 | 30.5  *N* = 71/233 | .5 | 22.8  *N* = 91/291 |

**Legend:** GA, gestational age; BPD, bronchopulmonary dysplasia; p-values for univariate analyses were derived from Chi square test.
